# The Prevalence of Growth Variations Among Pediatric Celiac Disease Patients at the Time of Diagnosis

**DOI:** 10.7759/cureus.11706

**Published:** 2020-11-25

**Authors:** Ghassan Sukkar, Alhusain M Alshareef, Marwan Aljahani, Hadeel A Alharthi, Abdulaziz Fakieha

**Affiliations:** 1 Pediatrics, King Saud Bin Abdulaziz University for Health Sciences College of Medicine, Jeddah, SAU; 2 Medicine, King Abdulaziz Medical City/King Saud bin Abdulaziz University for Health Sciences, Jeddah, SAU; 3 Faculty of Medicine, Ibn Sina National College, Jeddah, SAU

**Keywords:** tissue transglutamase, celiac disease

## Abstract

Background and aim

Celiac disease is an immune-mediated disorder caused by sensitivity to dietary gluten. Celiac patients typically present with malabsorption and low growth parameters; however, studies have shown that the presentation of celiac disease can have a higher percentage of patients with normal or high growth parameters and no signs of malabsorption. The study aims to estimate the prevalence of the growth variation found in children with biopsy-confirmed celiac disease at the time of diagnosis.

Methods

We included 31 biopsy-confirmed pediatric celiac patients diagnosed from 2007 to 2018 in King Abdulaziz Medical City, Jeddah, Saudi Arabia. Patients’ height, weight, and BMI at the time of diagnosis were converted to z-scores and growth percentiles according to the Centers for Disease Control and Prevention growth charts. In addition, patients’ comorbid conditions were also recorded.

Results

At the time of diagnosis, 45.16% of our patients presented as underweight, 41.94% of patients had normal weight, 6.5% were overweight and obese, respectively. The mean BMI was 15.44 (±3.65). Our population had a statistically significant lower BMI, height, and weight mean z-scores at the time of diagnosis.

Conclusion

A significant number of children diagnosed with celiac disease in our center had low weight, height, and BMI at the time of diagnosis. However, we emphasize that having normal growth parameters does not rule out the diagnosis of celiac disease.

## Introduction

Celiac disease (CD) is a chronic immune-mediated inflammatory disorder of the small bowel caused by sensitivity to dietary gluten as well as other related prolamins in individuals who are genetically susceptible [1]. The prevalence of celiac disease is estimated to affect 0.5 to one percent of the general population in many countries. In the United States, Australia, and Europe, the prevalence is estimated to range from 1:80 to 1:300 children [2]. Locally, according to a meta-analysis conducted by Safi MA, CD prevalence in Saudi Arabia is 1.4 percent [3]. This condition is characterized by both intestinal and extraintestinal manifestations, including diarrhea, dermatitis herpetiformis, anemia, short stature, and poor weight gain [4, 5]. The diagnosis of celiac disease is supported by positive serological tests such as tissue transglutaminase antibodies (tTG) and anti-endomysial antibodies (EMA). However, a small intestinal mucosal biopsy remains the gold standard for establishing a diagnosis [6]. The only effective treatment currently available for celiac disease is a strict life-long gluten-free diet (GFD) [2, 7]. The typical form of celiac disease is characterized by failure to thrive, malnutrition, and weight loss, especially in young children [8, 9]. However, over time the presentation of celiac disease has changed. Despite the known typical presentation of the disease, many studies show that the condition may also be associated with normal weight or overweight [5]. In a cohort study conducted in the United States, the percentage of normal weight and overweight patients was significantly higher than underweight patients at the time of diagnosis [10]. This implies that malnutrition is not necessarily always present at the time of diagnosis. Our aim in this study is to estimate the growth variation found in children diagnosed with celiac disease via biopsy at the time of the diagnosis.

## Materials and methods

We conducted a single-center cross-sectional chart review on pediatric celiac patients from 2007 to 2018 at King Abdul-Aziz Medical City (KAMC), Jeddah, Saudi Arabia. We included all patients aged from two to 14 years with biopsy-confirmed celiac disease diagnosis according to the Marsh criteria. Patients with genetic diseases that affect growth, and patients receiving growth-enhancing medications were excluded from the study. We used Raosoft software (Roasoft, Inc., US) to calculate our sample size. We used a margin of error of 5% and a confidence interval of 95%. The recommended sample size was 296. Patients’ height, weight, and BMI were converted to age-specific percentiles and z-scores derived from growth charts published by the Centers for Disease Control and Prevention (CDC). Additional information regarding the patients’ presentation, comorbidities, and serological markers were also collected. SAS software 9.4 (SAS Institute Inc., Cary, US) was used for statistical analysis. Categorical variables were described as frequencies and percentages, and continuous variables were described as mean and standard deviation. T-test was used to compare the means of the z-scores, Mann-Whitney test was used to compare two independent groups.

## Results

**Figure 1:**
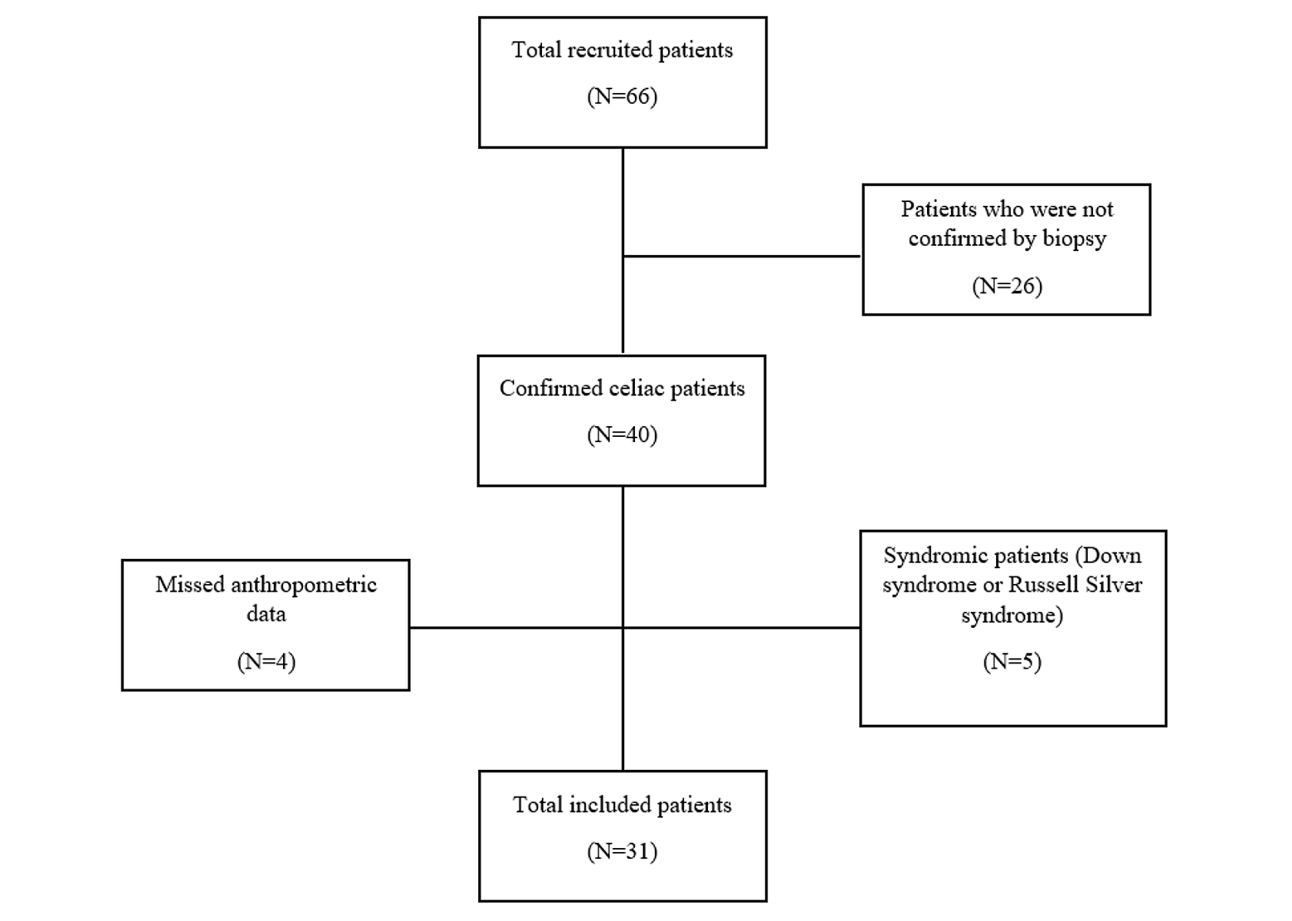
Patients selection process flowchart

**Figure 2:**
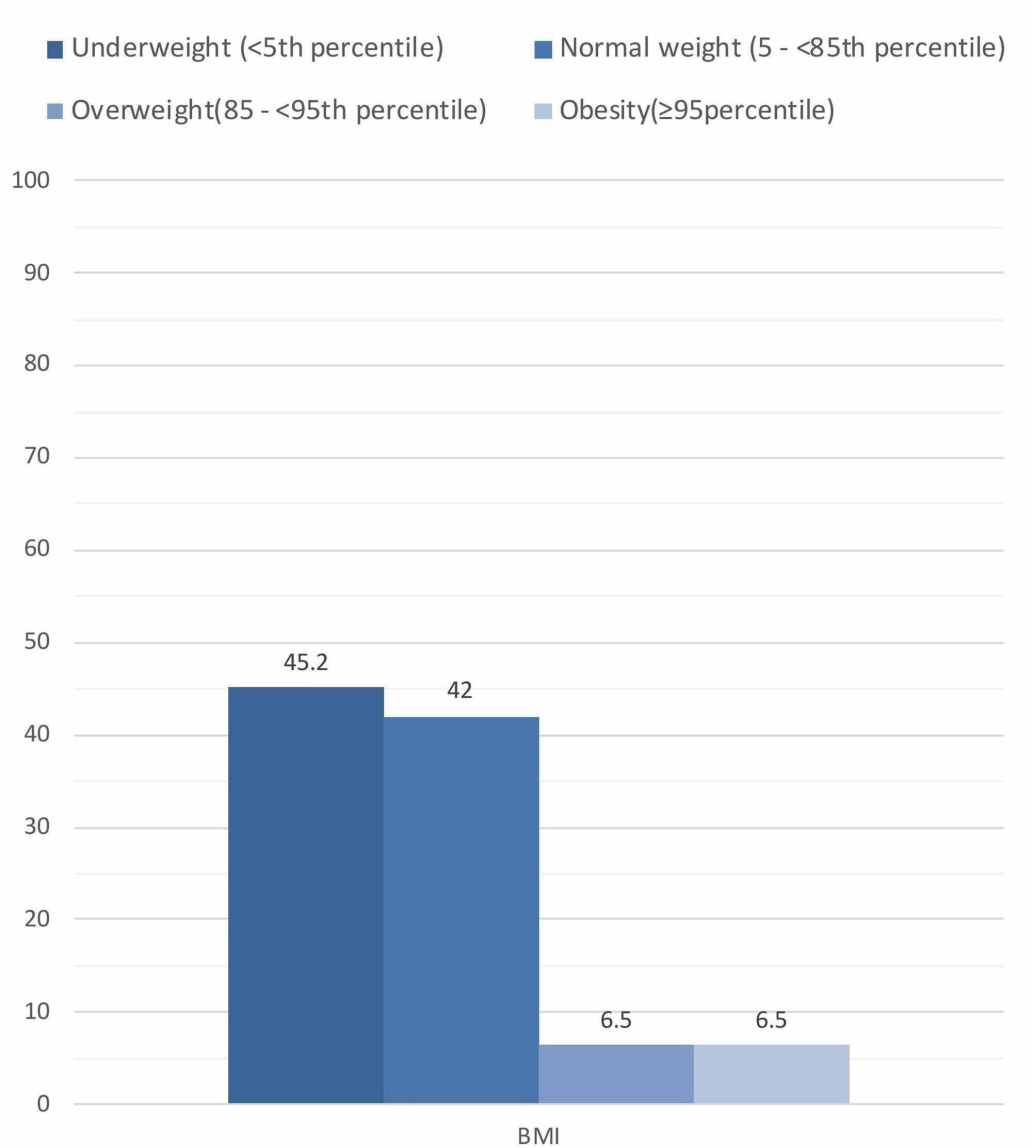
Percentages of BMI categories

**Figure 3:**
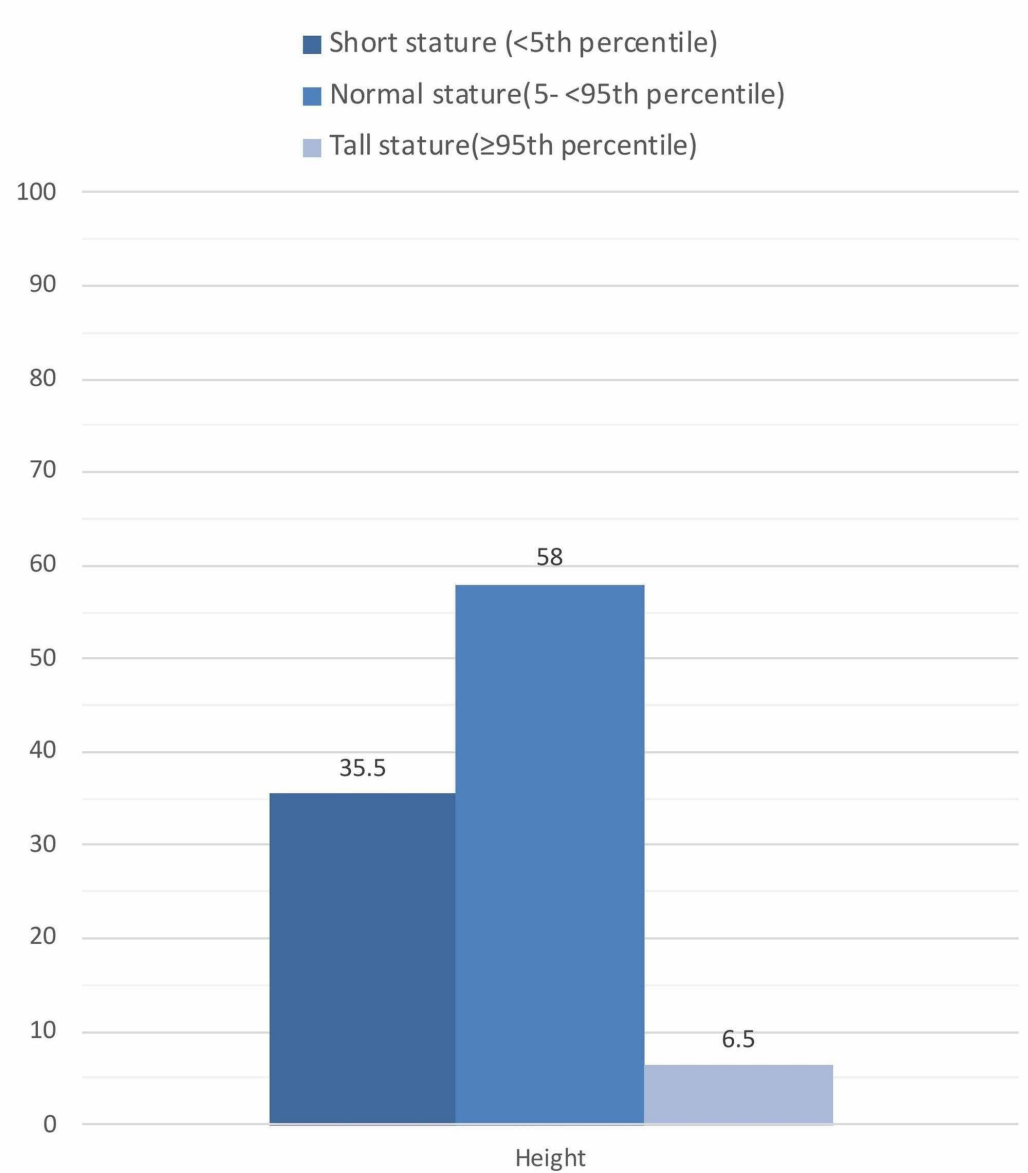
Percentages of height categories

Out of 66 patients, 31 patients fulfilled the research selection criteria (Figure 1). Their demographics and clinical characteristics are presented in Table 1. Nineteen (61.29%) were males, and 12 (38.71%) were females. The mean age was 7.71 (±3.09). The mean weight, height, and BMI were: 23.45 (±12.18), 119.77 (±18.27), 15.44 (±3.65), respectively. Their corresponding z-scores were: -1.37 (±1.80), -0.92 (±1.46) and -1.28 (±1.93). Fourteen (45.16%) patients were underweight, 13 (41.94%) were normal weight, two (6.45%) were overweight, and two (6.45%) were obese (see Figure 2). In terms of height, 18 (58.06%) had normal stature, 11 (35.48%) were short stature, and two (6.45%) were tall stature (see Figure 3). Nineteen (61.29%) patients were associated with different comorbidities in the following frequencies: diabetes mellitus type 1 (DMI) - nine (29.03%), asthma - five (16.12%), anemia - two (6.45%), attention deficit hyperactivity disorder (ADHD) - two (6.45%), and autism - one (3.22%). The mean anthropometric measures of CD patients were significantly different from the norm (z-score of zero) with p values of 0.0002 for mean weight, 0.0016 for mean height, and 0.0004 for mean BMI (Table 2). On the other hand, no statistical differences in these measurements were found when we compared comorbid patients with non-comorbid ones since the p-values were 0.62 for mean weight, 0.73 for mean height, and 0.73 for mean BMI (Table 3).

**Table 1:** Patients' demographic and clinical characteristics ADHD - attention deficit hyperactivity disorder

Characteristic	n=31
Mean age (±SD)	7.71 (±3.09)
Gender	Male: 19 (61.29%)
	Female: 12 (38.70%)
Mean weight at diagnosis (±SD)	23.45Kg (±12.18)
Mean height at diagnosis (±SD)	119.77cm (±18.27)
Mean BMI at diagnosis (±SD)	15.44 (±3.65)
Associated co-morbidities	
Diabetes mellitus type I	9 (29.0%)
Asthma	5 (16.1%)
Hereditary anemia	2 (6.5%)
ADHD	2 (6.5%)
Autism	1 (3.2%)

**Table 2:** Anthropometric measurements of patients at the time of diagnosis * T-test: compared to the normal population mean z-score (0).

Anthropometric measurements	Percentile	Z-score	P-value (*)
Mean weight for age (±SD)	25.32 (±34.79)	-1.37 (±1.80)	0.0002
Mean height for age (±SD)	30.69 (±31.38)	-0.92 (±1.46)	0.0016
Mean BMI for age (±SD)	28.74 (±34.01)	1.28 (±1.93)	0.0004

**Table 3:** Mean z-score of anthropometric measurements comparison between non-comorbid and comorbid patients

Anthropometric measurements	Non-comorbid	Comorbid	P-value
Mean weight for age z-score (±SD)	-1.50 (±1.71)	-1.29 (±1.90)	0.62
Mean height for age z-score (±SD)	-1.00 (±1.47)	-0.88 (±1.49)	0.73
Mean BMI for age z-score (±SD)	-1.37 (±1.67)	-1.23 (±2.13)	0.73

## Discussion

In this single-center retrospective review study, we reviewed the anthropometric measures of 31 biopsy-confirmed celiac patients to estimate their growth variations. Results showed that 45.16% of included patients were underweight at the time of diagnosis, 41.9% were normal weight, 6.5% were overweight, and 6.5% were obese. Additionally, 35% of patients had short stature at the time of diagnosis. 

Our findings in terms of weight categories were consistent with the classical belief that CD presents with malabsorption. In 2004, an American study conducted by Hoffenberg EJ et al. showed that children with seropositive CD had significantly lower z-scores for weight, height, and BMI compared to other children [11]. More recently, a study conducted in Italy found that the frequency of overweight and obesity at the time of CD diagnosis was notably reduced compared to the general population. This reduction persisted even during the application of the gluten-free diet [12]. Some studies link this decrease in BMI to other factors such as the extent of the mucosal injury, diarrhea at the time of diagnosis, and female sex [13].

On the contrary, in the United States, Reilly et al. found that the majority of their celiac patients (142) had a normal BMI at the time of diagnosis (74.5%), and more patients were found to be overweight (19%) than underweight (6.5%) [10]. It is important to note that their study had a larger sample size, explaining why our results are different. Locally at King Abdulaziz University, a retrospective study was conducted on 80 celiac pediatric patients to describe the clinical pattern of the disease. The study found that only 23% of patients were underweight at the time of diagnosis, while the majority of their patients presented with a normal BMI. However, the study also showed that 38% of their patients had short stature at the time of diagnosis, which is very similar to our research finding (35%) [14].

Even though our results generally show that our patients are significantly underweight, it is important to note that having a normal or high BMI does not rule out the possibility of celiac disease. This point is reaffirmed in our findings since 54.9% of our patients had normal or high BMI at the time of diagnosis. 

The pathogenesis behind the atypical weight presentation of celiac disease is explained by the “compensatory” hypothesis [5]. The damaged atrophic villi are countered by compensatory mechanisms in the distal, functionally preserved parts of the intestines. An increase in the number of epithelial cells, the height of villi and the depth of the crypt cells compose the morphological adaptations that will increase nutrient absorption level [5]. Hypothetically, when these measures overcompensate the proximal villous damage, it may lead to an excessive caloric extraction and thus leads to an overweight or obese presentation [5]. The presence and severity of CD's classical symptoms are principally affected by the capability and efficiency of the compensatory mechanisms rather than the degree of villous atrophy or the extent of intestinal insult [15, 16]. However, since these adaptations are time-dependent, they increase as age increases, which may explain the tendency of atypical presentation in older children and adolescents [5].

Regarding the reliability of the presence of malabsorption for clinical suspicion of CD, van der Pals et al. conducted a study with a large sample of 12,632 12-year-old children screened for CD, and their anthropometric measures were recorded at the time of diagnosis. The study concluded that children with CD had lower weight, height and BMI compared to children with no CD, yet at an individual level, their growth parameters were not a reliable input in the diagnosis of CD [8].

CD and type 1 diabetes share a common genetic and possibly an environmental basis for their pathogenesis [17]. Therefore, the two conditions frequently present together. In fact, it is estimated that the global risk of CD complicating type 1 diabetes ranges from 2.5% to 16.4 [18]. This supports our results since 29% of our CD patients had type 1 diabetes. However, our results show no significant differences in the z-scores of any of the anthropometric measures between non-comorbid and comorbid (including Type 1 diabetes) groups. On the contrary, Tsouka et al. study showed that children with CD and T1DM had significantly higher anthropometric measures and a lower incidence of anemia compared to children with CD alone [19]. This might be attributed to the anabolic effect of insulin therapy [20]. 

Limitations to our research include missing data from patient files due to the retrospective nature of our study. Moreover, our research had a small sample size, which can be attributed partly to many files being missing or lost during the transition of paper files to the new electronic medical record system in King Abdulaziz Medical City. We also used the CDC growth charts, which might not represent our population accurately.

## Conclusions

Based on our findings, we concluded that celiac patients in our center have a significantly lower BMI, weight for age, and height for age at the time of diagnosis. We also conclude that having normal or high growth parameters does not exclude the possibility of celiac disease. Our recommendations for this topic's future research is to increase the sample size by including more patients from other centers in our region. We also recommend measuring the effectiveness of the gluten-free diet on the growth parameters of celiac patients and the progression of the condition. Finally, we suggest using a customized growth chart that is more representative of our local population.
